# Integrated transcriptional profiling and genomic analyses reveal *RPN2* and *HMGB1* as promising biomarkers in colorectal cancer

**DOI:** 10.1186/s13578-015-0043-9

**Published:** 2015-09-17

**Authors:** Jialing Zhang, Bin Yan, Stephan Stanislaw Späth, Hu Qun, Shaleeka Cornelius, Daogang Guan, Jiaofang Shao, Koichi Hagiwara, Carter Van Waes, Zhong Chen, Xiulan Su, Yongyi Bi

**Affiliations:** School of Public Health, Wuhan University, Wuhan, China; Clinical Medicine Research Center, The Affiliated Hospital, Inner Mongolia Medical University, Hohhot, China; Tumor Biology Section, Head and Neck Surgery Branch, National Institute on Deafness and Other Communication Disorders, NIH, Bethesda, USA; Laboratory for Food Safety and Environmental Technology, Shenzhen Institutes of Advanced Technology, Chinese Academy of Sciences, Shenzhen, China; Pediatric Endocrinology Unit, Department of Women’s and Children’s Health, Karolinska Institute and University Hospital, Stockholm, Sweden; Department of Oncology, The Affiliated Hospital, Inner Mongolia Medical University, Hohhot, China; Department of Biology, Hong Kong Baptist University, Hong Kong, China; Department of Bioinformatics, School of Basic Medical Sciences, Nanjing Medical University, Nanjing, China; Department of Respiratory Medicine, Saitama Medical Center, Jichi Medical University, Saitama, Japan

**Keywords:** Colorectal cancer, Gene signature, Genomic alterations, Transcription factor, Gene regulation

## Abstract

Colorectal cancer (CRC) is a heterogeneous disease that is associated with a gradual accumulation of genetic and epigenetic alterations. Among all CRC stages, stage II tumors are highly heterogeneous with a high relapse rate in about 20–25 % of stage II CRC patients following surgery. Thus, a comprehensive analysis of gene signatures to identify aggressive and metastatic phenotypes in stage II CRC is desired for a more accurate disease classification and outcome prediction. By utilizing a Cancer Array, containing 440 oncogenes and tumor suppressors to profile mRNA expression, we identified a larger number of differentially expressed genes in poorly differentiated stage II colorectal adenocarcinoma tissues, compared to their matched normal tissues. Ontology and Ingenuity Pathway Analysis (IPA) indicated that these genes are involved in functional mechanisms associated with several transcription factors. Genomic alterations of these genes were also investigated through The Cancer Genome Atlas (TCGA) database, utilizing 195 published CRC specimens. The percentage of genomic alterations in these genes was ranked based on their mRNA expression, copy number variations and mutations. This data was further combined with published microarray studies from a large set of CRC tumors classified based on prognostic features. This led to the identification of eight candidate genes including *RPN2*, *HMGB1*, *AARS*, *IGFBP3*, *STAT1*, *HYOU1*, *NQO1* and *PEA15* that were associated with the progressive phenotype. In particular, *RPN2* and *HMGB1* displayed a higher genomic alteration frequency in CRC, compared to eight other major solid cancers. Immunohistochemistry was performed on additional 78 stage I–IV CRC samples, where RPN2 protein immunostaining exhibited a significant association with stage III/IV tumors, distant metastasis, and poor differentiation, indicating that RPN2 expression is associated with poor prognosis. Further, our study revealed significant transcriptional regulatory mechanisms, networks and gene signatures, underlying CRC malignant progression and phenotype warranting future clinical investigations.

**Electronic supplementary material:**

The online version of this article (doi:10.1186/s13578-015-0043-9) contains supplementary material, which is available to authorized users.

## Background

Colorectal cancer (CRC) is the third most common cancer worldwide [1]. TNM staging is a standard pathology classification used for treatment strategy and outcome prediction, especially for most stage-I, -III, and -IV CRC patients. In the clinical setting, approximately 90 % of localized stage I CRC patients are cured by surgical removal of the tumor burden, so the prognosis and treatment plan for stage I CRC patients has been standardized [2, 3]. However, stage II CRC is highly heterogeneous, with 20–25 % of patients exhibiting recurrence or relapsed disease following surgery. The 5-year overall-survival of patients with stage II tumors ranges from 58 to 85 % [4–6]. Although a wide variety of potential clinical and pathological risk factors have been examined for improved outcome prediction, such as T4 lesions, poorly differentiated histology or intestinal obstruction [7], the molecular mechanisms underlining the heterogeneous characteristics of stage II CRC are still not well established. In fact, previous publications indicated that clinical outcome prediction and treatment of stage II CRC remains controversial, with a necessity for a better molecular classification utilizing gene signatures and biomarkers, in order to complement TNM staging [8–10].

Over the past few years, there has been a significant progress in identifying distinct molecular signatures to better define CRC subsets. The biological and clinical significance of overexpressed oncogenes (i.e. *EGFR* and *MYC*), and functional loss of tumor suppressor genes (i.e. *TP53* and *APC*) have been well characterized [11–15]. Better understanding of the oncogenes and related signaling pathways have led to successful CRC therapies, especially in targeting the *EGFR*-*RAS*-*MAPK* signaling pathway [16, 17]. Further discoveries on TP53 and APC have been utilized to predict poor CRC prognosis, with the presence of defective of APC expression or point mutations in *TP53* [18]. However, these gene signatures have not been successfully utilized as biomarkers to classify the heterogeneous stage II CRC for diagnosis and treatment. More comprehensive gene signatures and signaling transduction pathways related to stage II CRC are needed to understand the disease progression and for an improved prognosis, as well as treatment.

Recent development of high throughput technologies, such as gene expression profiling and genomic sequencing analysis, enabled us to identify comprehensive cancer gene signatures and related signaling pathways, based on the genetic and expression alterations in multiple cancers [19, 20]. Previously published gene signatures using gene profiling, RT-PCR, or sequencing technologies varied considerably in terms of their gene composition, with little gene overlap [21]. The lack of concordant gene signatures could be related to several issues, including differences: (1) in technological platforms, such as microarray, RT-PCR or sequencing technologies; (2) different sample types selected for analysis, and (3) the different analytical tools used to generate the gene signatures [22]. Hence, an integrative approach combining the information derived from different technological platforms, summarizing different categories of genetic and expression alterations from a large number of samples, may more accurately identify the associations of clinical phenotypes with genetic and expression alterations.

In the current study, we performed an integrated data analysis, combining gene expression profiles from our collected paired stage II CRC patient’s samples, with genomic alteration data of 195 CRC samples, previously published by the Cancer Genome Atlas (TCGA) project, along with extracted results from more than 50 previously published microarray studies. This integrated approach has identified eight gene candidates, significantly associated with a progressive CRC phenotype. Among these genes, a significantly higher alteration profile for *Ribophorin II* (*RPN2*) and *High*-*mobility group protein B1* (*HMGB1*) was observed in CRC tumors, compared to other eight major human solid cancer types, currently available in the TCGA database. Immunohistochemistry was performed on 78 clinical stage I–IV CRC samples, where RPN2 exhibited a significant association with distant metastasis and poor differentiation. These gene signatures expand the current CRC biomarker pools for tumor progression and CRC outcome prediction. Further, these gene signatures warrant future validation as potential biomarkers in large clinical trials.

## Results

### Identification of gene expression signatures, related pathways and upstream regulators

**Fig. 1:**
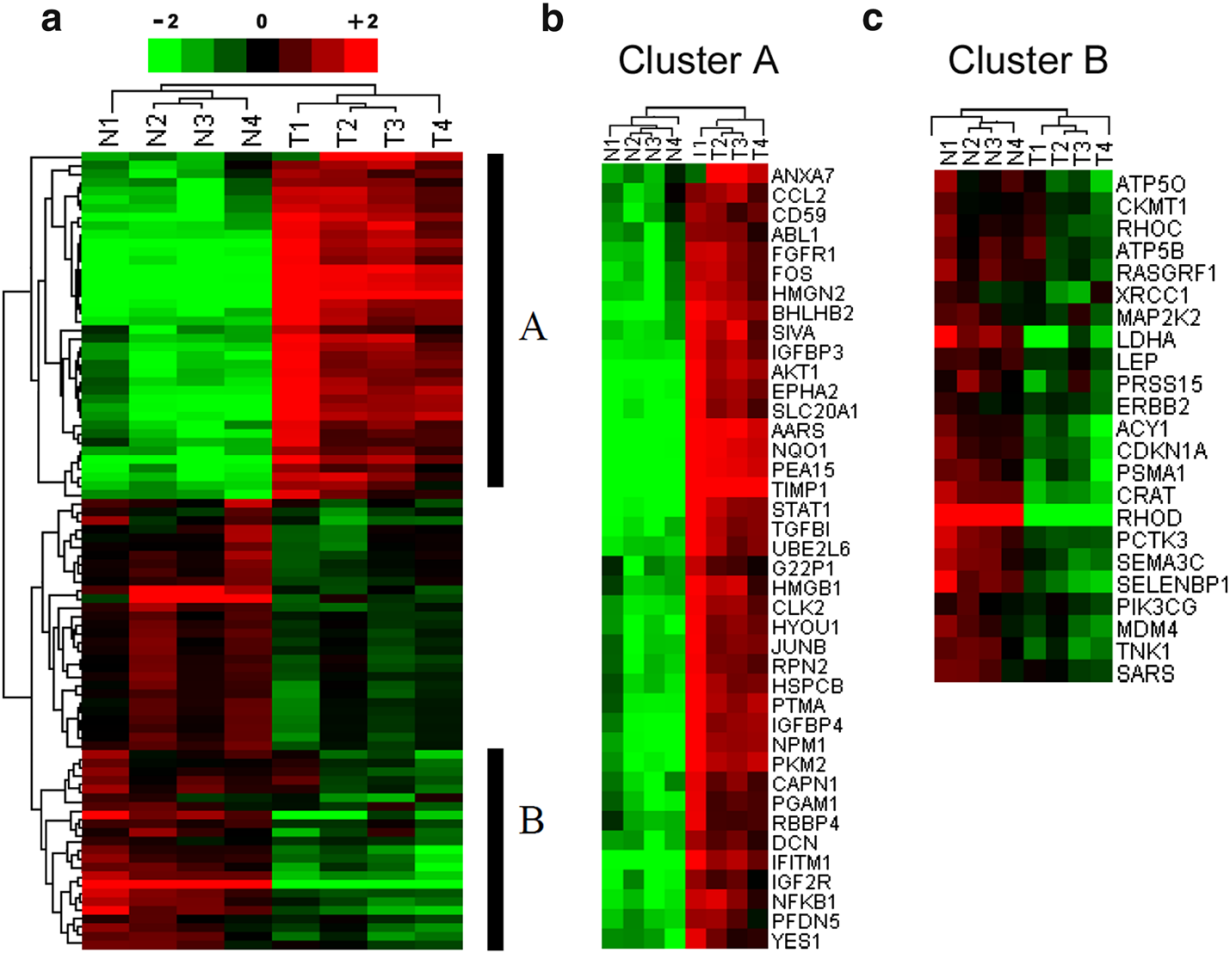
Identification of CRC gene signatures using global expression profiles. Microarray analysis was performed using stage II CRC cancer specimens (T1-4), compared to matched mucosal tissue samples (N1-4). **a** Unsupervised hierarchical clustering of differentially expressed genes was used to identify differentially expressed genes (fold change 1.5, p < 0.05). Over-expressed and under-expressed genes are indicated by *red* and *green* colour. The expression level is proportional to colour brightness. *Black bars* on the *left* indicate gene *clusters A* and *B*, respectively. An expanded view of *cluster A* (over-expressed genes, **b**), or *cluster B* (under-expressed genes, **c**), with the indicated gene names is shown

To investigate important gene signatures in stage II CRC tissues with implications in aggressive malignant phenotypes, we utilized a cancer-specific array, containing known cancer related gene probes (N = 440) for mRNA expression profiling. A total of 92 genes, exhibiting at least 1.5-fold difference with p < 0.05 in mRNA level between tumor and normal samples were identified (Additional file 1: Table S1). Unsupervised hierarchical clustering algorithm was used to classify sub-populations based on the list of differentially expressed genes. As expected, tumor and normal samples were clearly separated into two subgroups (Fig. 1a). We further identified two characteristic clusters, including an over-expressed gene cluster A and an under-expressed gene cluster B in the tumor subgroup (Fig. 1b, c).

To investigate their functional relevance, we annotated differentially expressed genes in Fig. 1 according to Gene Ontology (GO) biological processes, by using the DAVID software. We observed significant enrichment in apoptosis, phosphorylation, cell proliferation, protein kinase cascade, colorectal cancer metastasis and intracellular signaling cascade in stage II CRC (Additional file 1: Table S2a). We further applied IPA (Ingenuity Pathway Analysis) to identify enriched pathways and unveil the functional relevance of our differentially expressed genes in stage II CRC. Using this approach, six enriched sub-networks were hence identified. They were associated with NFKB, AP1, STAT3, TP53, HSP90 and CTNNB1 signaling pathways (Additional file 2: Figure S1). Furthermore, utilizing the IPA tool, we also investigated upstream regulatory molecules that are responsible for identified pathways and altered gene expression in stage II CRC. As shown in Additional file 1: Table S2b, three top transcription regulators TP53, TP63 and TP73 were significantly enriched in stage II CRC. In addition, several transcription factors and oncogenes ranked top on the list, such as NFKB, AP1, STAT3 and MYC, as well as other regulators (i.e. E2F1, HIF1A and ANR). This data suggests that aberrant regulation of these TFs (NFKB, AP1, STAT3, TP53, TP63 and MYC) could potentially influence the stage II CRC progression.

### Transcriptional regulatory gene network in CRC

**Fig. 2:**
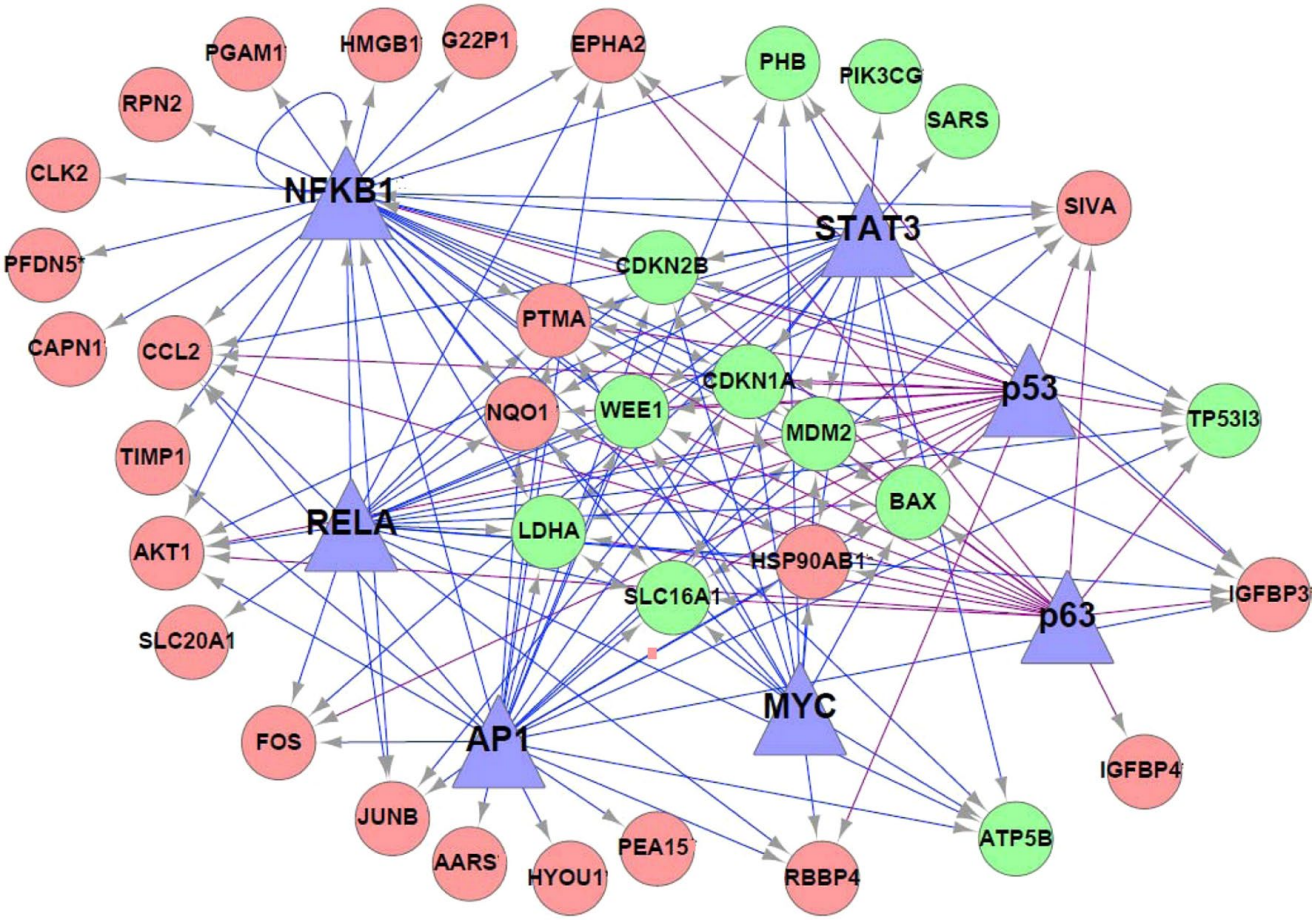
Inferred transcriptional regulatory gene network in CRC. A newly developed computational model was utilized to identify target genes of seven cancer-related TFs and to construct gene regulatory networks. The *triangular nodes* represent corresponding TFs. *Circle nodes* refer to the target genes of TFs. *Arrow lines* show regulatory relationships from TFs to their target genes. *Purple* or *blue lines* stand for TFs that act as tumor suppressor or oncogene, respectively. *Red* and *green* nodes refer to over- and under-expressed genes, respectively

To test the hypothesis on how upstream oncogenic and tumor suppressor TFs regulate gene expression in stage II CRC, we applied a previously developed bioinformatics model [24] able to predict these TFs regulation of their target genes. As shown in Additional file 1: Table S1, we generated a list of putative targets for the seven TFs, including NFKB1, RelA, TP53, TP63, STAT3, MYC and AP1. About 35–40 % of NFKB1 or TP53 targets were previously validated by experimental data (Additional file 1: Table S1). There were twenty-nine NFKB1 targeted genes, consisting of both predicted and validated gene candidates, as well as eight unique NFKB1 target genes, including RPN2 and HMGB1 (Additional file 1: Table S1). In addition, there were many genes under the regulation of multiple TFs (Additional file 1: Table S1). Subsequently, we constructed a transcriptional regulatory gene network, presented in Fig. 2. We predicated ten genes being co-targeted by all of the seven TFs, including under-expressed (*BAX*, *CDKN1A*, *CDKN2B*, *LDHA*, *MDM2*, *SLC16A1*, *WEE1*) and over-expressed *(HSP90AB*, *NQO*1 as well as *PTMA*) genes, respectively. These genes are known to be involved in biological processes, such as apoptosis, cell proliferation, and cell cycle. Our data suggests that the interaction of these seven TFs may participate alone or co-regulate the signaling pathways, associated with the progression of stage II CRC.

### Genomic and expression alterations of identified CRC-related genes in the TCGA database

**Fig. 3:**
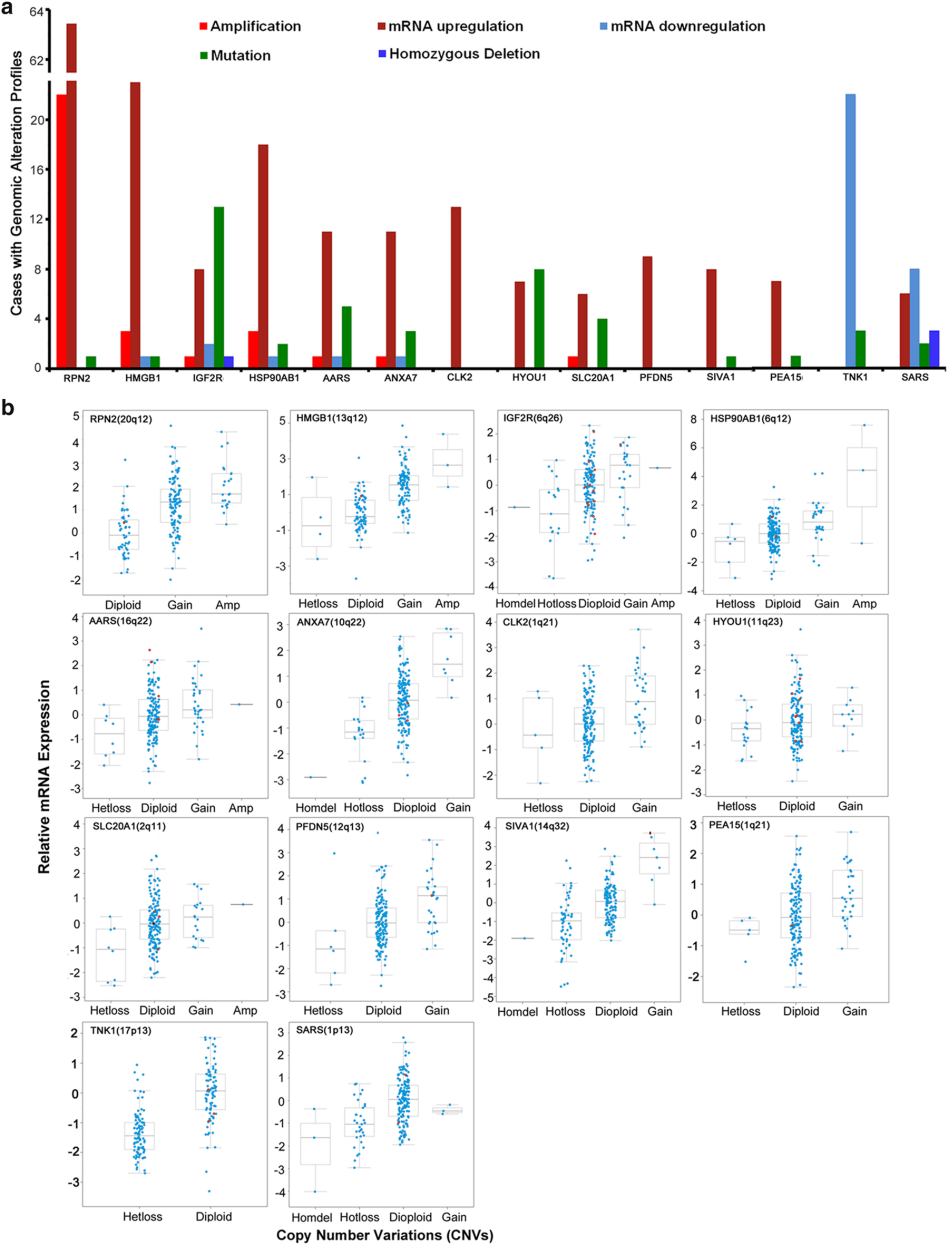
Evaluation of genomic alterations in TCGA CRC dataset. **a** Genomic alterations, including copy number variations (CNVs), mutations and gene expression of each gene candidate were extracted from the TCGA colorectal cancer (CRC) database. The *X*- and *Y*-axis represent genomic alterations and case number, respectively. **b** Variations of mRNA expression versus CNVs and chromosomal locations for individual gene candidates are shown. CNV categories include homozygous deletions (Homodel), heterozygous deletions (Hetloss), diploid, gain and amplification (Amp). mRNA was expressed as 25th, 50th, and 75th percentile and *whiskers* represent minimal and maximal values, excluding the outliers. *Red circle* indicates missense mutations

To identify the genomic alterations for the differentially expressed genes discovered in this project, we took the advantage of the TCGA database, containing recently published large mRNA expression and genomic alteration data, derived from 195 stage I–IV CRC patients [20]. We analyzed 92 differentially expressed genes identified in our study, using the TCGA database and determined that a total of 24 genes (22 over- and 2 under-expressed genes), exhibited consistent expression patterns with the TCGA data (Additional file 2: Figure S2). Among the over-expressed genes, *RPN2* was altered most frequently in 87 out of 195 (37 %) CRC cases at stage II and III, including significant gene amplification and mRNA overexpression. The second most altered gene was *HMGB1* (13 %), which exhibited a significantly higher rate of mRNA up-regulation (Fig. 3a; Additional file 2: Figure S3). The remaining 19 over-expressed genes were altered similarly in 195 CRC cases (5–10 %) and across all stages (Fig. 3a; Additional file 2: Figure S3a). Alterations of *TNK1* included mRNA down-regulation or mutations in 22 out of 195 cases (13 %) across all tumor stages (Additional file 2: Figure S3). We further examined if copy number variations (CNVs) are associated with mRNA expression for the 24 genes, identified in the TCGA. The mRNA expression of 14 out of these 24 genes correlated significantly with CNVs (Fig. 3b). In addition, ≥25 % recurrent CNVs were observed as gains on chromosomes 20q, 13q, 6q, 16q, 10q, 11q, 12q, 14q, and 1q, as well as losses at 17p and 1p, respectively (Fig. 3b). Remarkably, *RPN2* and *HMGB1* had a higher percentage of CNV in these cases, including gains (*RPN2* and *HMGB1*) and amplifications (RPN2, Fig. 3b).

### Identification of CRC-specific gene signatures across multiple data sets

Previous evaluation of 29 microarray studies, utilizing CRC tumor specimens, has identified 31 gene signatures with prognosis significance [31]. Out of these 31 genes, 28 were overlapping with our identified differentially expressed genes from our microarray data, of which 8 genes (*AARS*, *PEA15*, *NQO1*, *STAT1*, *IGFBP3*, *HYOU1*, *HMGB1* and *IGF2R*) were also matched genes, previously verified in the TCGA dataset (Table 1; Fig. 3; Additional file 2: Figure S3). Next, we manually searched for the presence of these 28 differentially expressed genes in 50 previously published microarray studies that were utilizing CRC tissues (Additional file 1: Table S3). The 50 published microarray studies were selected based on prognosis signature inclusion criteria. Sixteen out of these 28 genes were overlapping with the 50 published microarray experiments. Further investigation revealed that 10 out of these 16 genes (*RPN2*, *AARS*, *PEA15*, *NQO1*, *AKT1*, *STAT1*, *IGFBP3*, *HYOU1*, *HMGB1* and *IGF2R*) also matched with the identified genes in the TCGA database (Table 1).

**Table 1:** Comparison of differentially expressed genes with published CRC microarray studies

Gene symbol	Fold change^a^	Tumors with prognosis gene signatures^b^	Tumors from other microarrays^c^
TIMP1	8.34	232	52
AARS^d^	4.48		30
PEA15^d^	4.23	215	22
CD59	1.61	232	
IFITM1	3.03		106
HMGB1^d^	2.07	215	496
RPN2^d^	1.91		15
IGF2R	1.75	215	107
IGFBP3^d^	2.29	232	706
NPM1	2.60	87	679
NQO1^d^	3.77	55	200
TGFB1	2.35		531
AKT1	3.33		43
HYOU1^d^	2.16		30
STAT1^d^	2.83	215	30

^a^The fold changes of differential gene expression between Stage II CRC tumor and normal tissues were extracted from the microarray data presented in Fig. 1

^b^Prognosis gene signatures were extracted from the publication of Sanz-Pamplona et al., which surveyed 29 microarray studies with patient’s data of recurrence, metastasis and survival [25]

^c^Additional microarray data from 50 previous publications was extracted from CRC tumor and metastatic specimens (Additional file 1: Table S3). The indicated number stands for tumor specimens with increased gene expression

^d^Refers to genes that are involved in transcriptional regulation (Fig. 3; Additional file 1: Table S1) and an overlapping with TCGA data (Additional file 2: Figure S2)

**Fig. 4:**
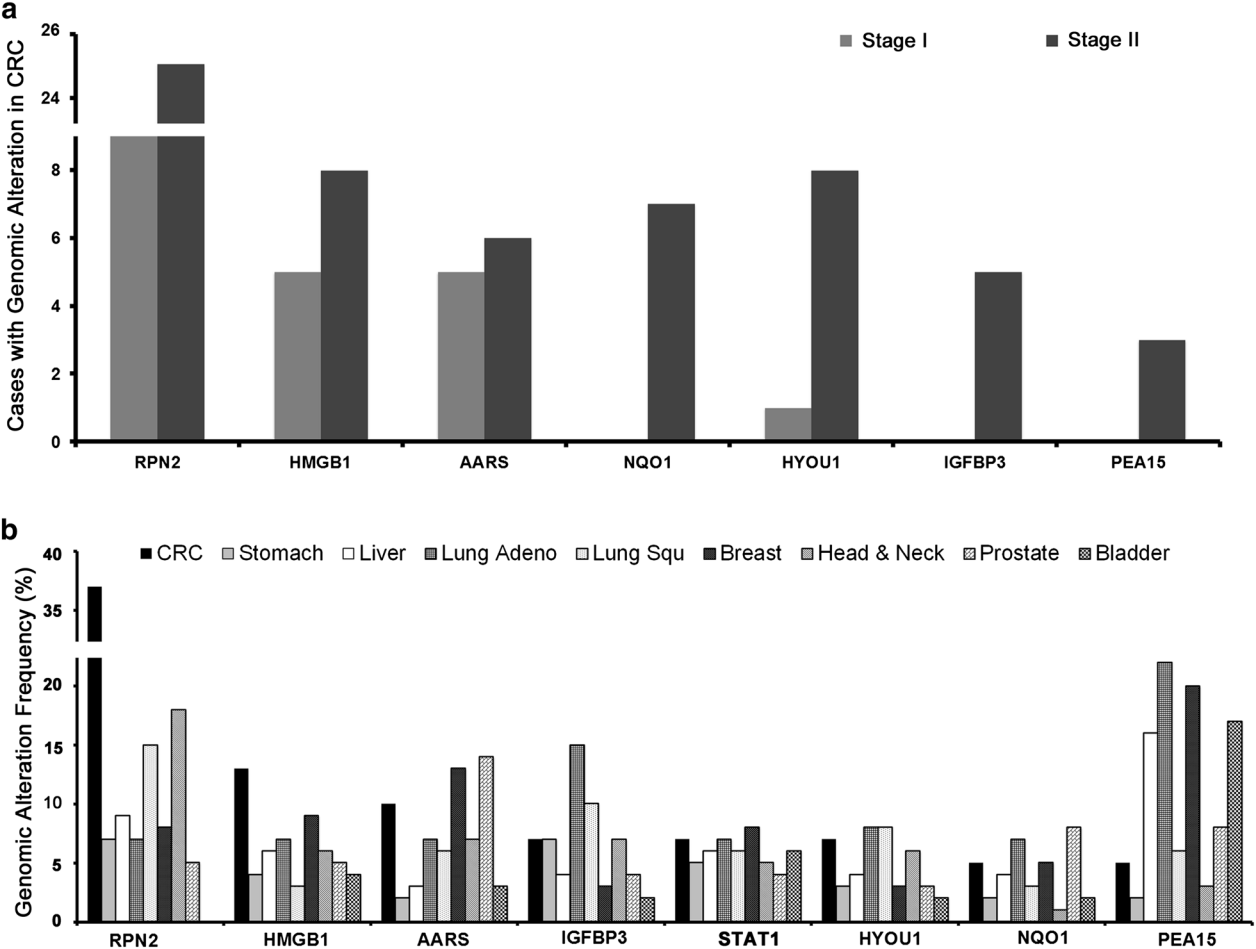
Identification of CRC-specific gene signatures across most common solid tumors. **a** Case numbers with genomic alterations for each gene candidate, identified in stage I/II colorectal cancer from the TCGA dataset. **b** The frequency of genomic alterations for each gene candidates was analyzed across nine different solid cancer types (each with >150 tumor samples) from the TCGA database

Finally, we selected 8 out of the above 10 genes from above list (*RPN2*, *HMGB1*, *AARS*, *IGFBP3*, *STAT1*, *HYOU1*, *NQO1* and *PEA15*), based on the following criteria: (1) previously identified by CRC tumor microarray experiments; (2) previously published as a prognosis markers and (3) identified candidate within the transcriptional regulation network presented in Fig. 2. The genomic alterations of these selected genes appeared to be more evident in stage II, compared to stage I CRC tumors, as observed in the TCGA database (Fig. 4a). Furthermore, we analyzed these eight genes for alteration frequency in nine major solid cancer types, available in the TCGA database. A significantly higher alteration frequency for *RPN2* and *HMGB1* genes was observed in CRC tumors, when compared with other tumors (Fig. 4b).

### Association of RPN2, HMGB1 and NFkB1 protein expression with CRC clinic-pathological features

**Fig. 5:**
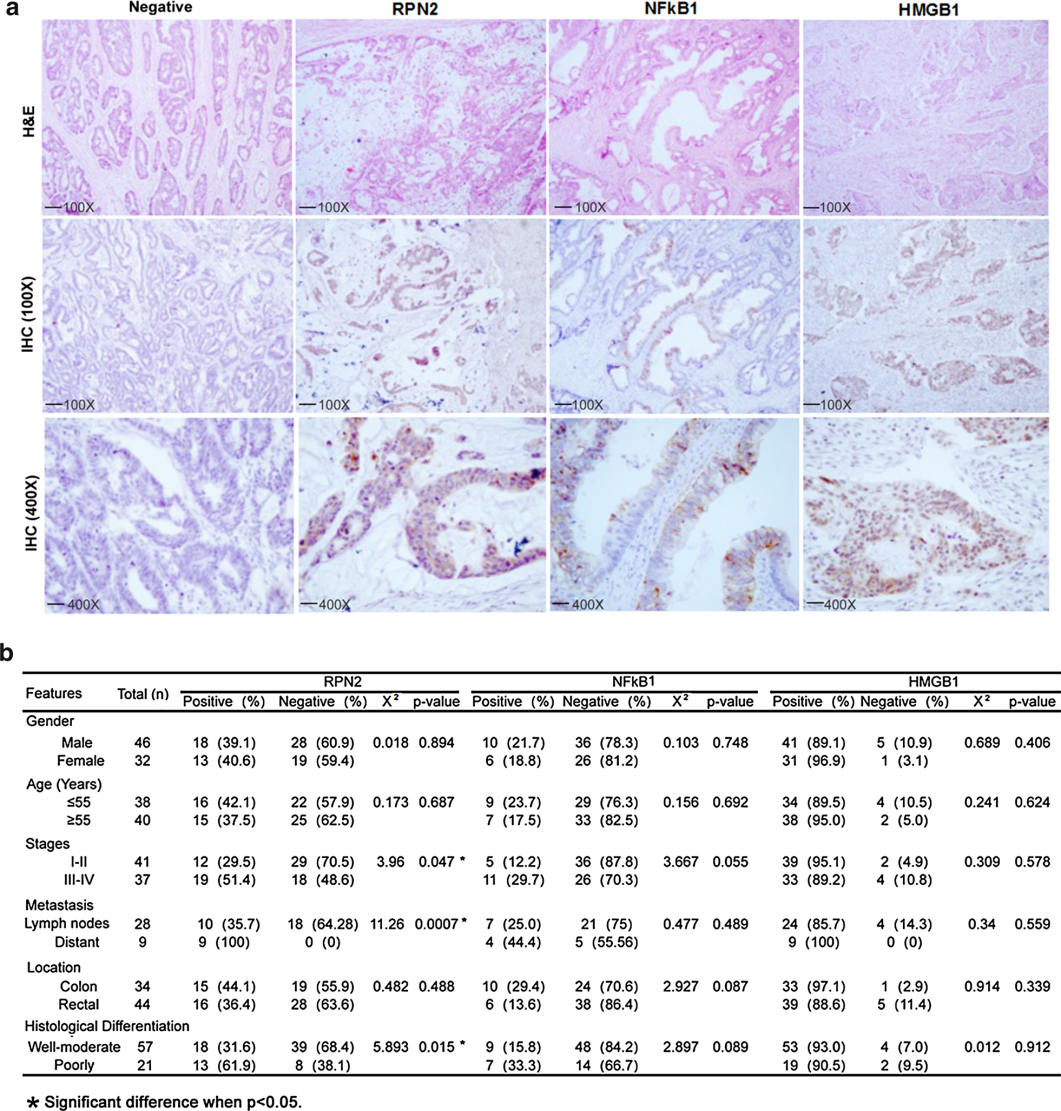
Association of RPN2, NFkB1 and HMGB1 protein expression with clinicopathological features in a cohort of CRC. **a** Immunohistochemical analysis of RPN2, HMGB1 and NFkB1 protein expression was performed in a cohort of additional 78 CRC specimens. H&E refers to Hematoxylin and Eosin staining. Microscope images were taken at either ×100 or ×400 magnifications. The *low-scale bar* represents 200 μm and *high-scale bar* corresponds to 50 μm. **b** Correlation analysis of RPN2, NFkB1 and HMGB1 expression by gender, age, stages, metastasis, tumor location, and histological differentiation in 78 CRC samples. The cases with metastasis were divided into lymph node only and distant metastasis. *Corresponds to a p value <0.05 (Fisher’s exact test)

Based on our observations, where a higher percentage of CRC cases exhibited an association between genetic alterations and expression profiles in *RPN2* and *HMGB1,* as well as *NFkB1*, a common transcriptional regulator of both genes, we performed immunohistochemistry to validate RPN2, HMGB1 and NFkB1 protein expression in a cohort of additional 78 CRC specimens (Fig. 5). A total of 29.5 % of stage I/II and 51.4 % of stage III/IV specimens were positive for cytoplasmic RPN2, with a significant association between tumor stages (p = 0.047). In addition, RPN2 staining was also strongly associated with distant metastasis (p = 0.0007) and histological differentiation (p = 0.015), but not with gender, age or tumor location (Fig. 5a, b). Characteristic cytoplasmic and nuclear staining was observed for NFkB1 in the majority of tumor specimens (stage I–IV). However, cases with positive staining were highest in stage III/IV tumors but barely reached statistical significance (p = 0.055, Fig. 5a, b). No significant association of NFkB1 protein expression was observed for distant metastasis or other clinic-pathological features (Fig. 5b). In addition, ~90 % of all examined CRC tumor samples exhibited HMGB1 immuno-reactivity, however no difference association with clinicopathological features was observed (Fig. 5b).

## Discussion

In this study, we performed an integrated data analysis combining differentially expressed genes from microarray (Fig. 1), with published literature (Additional file 1: Table S3) and available genomic, as well as expression datasets from TCGA (Figs. 2, 4). The integrated analysis identified novel gene signatures in stage II CRC tumors, with strong implications for late stage aggressive and metastatic phenotypes (Table 1; Fig. 5). Bioinformatics analysis indicated that these genes were regulated by seven TFs, including NFKB1, RelA, TP53, TP63, STAT3, MYC and AP1 (Fig. 3), and further enriched in NFKB, AP1, STAT3, TP53, HSP90 and CTNNB1 pathways, known to be important regulators for cell proliferation, cell cycle, apoptosis, and intracellular signaling (Additional file 1: Table S1; Additional file 2: Figure S1). Furthermore, integrated evaluation of large published CRC datasets from TCGA identified eight candidate genes that were associated with the progressive phenotype of CRC (Table 1; Fig. 4a). A significantly higher alteration frequency for *RPN2* and *HMGB1* was also observed in CRC tumors, compared to other eight common solid tumor types. Finally, immunohistochemistry of RPN2, HMGB1 and NFkB1, revealed a significant association of RPN2 with CRC stage, metastasis and differentiation, in a cohort of additional CRC samples (Fig. 5). Our data revealed an association of important gene signatures with aggressive stage II CRC and their underlining molecular regulatory mechanisms.

Among our differentially expressed gene list, we observed several overexpressed genes being tightly regulated by several critical TFs that control cell proliferation, survival and inflammation [32–34]. Using IPA analysis, to identify upstream regulators for the differentially expressed genes in stage II CRC samples used in this study, we observed a strong enrichment for tumor suppressor TP53 family members and oncogenic TFs (i.e.NFKB, AP1 and MYC) that either individually or combined regulate gene expression through shared or unique target genes (Fig. 2; Additional file 1: Table S1). The tumor suppressor TP53 family members and oncogenic TFs (NFKB1, AP1 and MYC) are thought to play a crucial role in controlling CRC progression, consistent with the observed results in the published TCGA report [20]. Our data suggested an existing strong link between the regulatory programs of these TFs in CRC. In particular, this study unveiled several potentially unique target genes for each TF, such as *RPN2* and *HMGB1* being targeted by NFKB1. These findings are consistent with previous studies of these important TFs in carcinogenesis [35–37]. Our experimental data from microarray is supported by both computational analysis and literature searches, where differentially expressed gene signatures are shown to promote the malignant CRC process.

Additional support for our experimental data comes from the analysis of gene signature expression profiles across 195 CRC samples that are associated with different CRC stages from the TCGA database (Additional file 2: Figure S2). We found that about 55 % of identified over-expressed genes from our cancer array did overlap with published TCGA data. Interestingly, several genes exhibited recurrent CNVs (frequency > 25 %), which directly modulate their mRNA expression (Fig. 3b), hence providing a genetic mechanism for gene expression, showing consistency with previous reports [38, 39]. These findings highlight our observation that this subset of overexpressed genes may play a critical role in genomic instability, which is significantly associated with progressive CRC phenotypes. We further examined gene signatures with a prognostic CRC marker potential and by utilizing an integrated analysis approaches, we have identified eight gene candidates, including *RPN2*, *HMGB1*, *AARS*, *IGFBP3*, *STAT1*, *HYOU1*, *NQO1* and *PEA15*. Among this list, six out of eight genes have been previously implicated in deregulation of gene expression and associated with the prognosis of CRC and other cancer types [40–43]. Only *RPN2* and *AARS* were novel genes, with no previous publication describing their functional contribution to CRC. The genetic alteration and expression profiles for this eight candidate genes were compared across different cancer types. Only *RPN2*- and *HMGB1*-genes were found to be most altered in CRC (Fig. 4b), further supporting their biological significance in CRC pathogenesis. We used a cohort of 78 independent CRC specimens (stages I–IV) to validate RPN2, HMGB1 and NFkB1 protein expression and their association with several clinic-pathological features. We observed that only RPN2 expression was significantly associated with tumor stage, histological differentiation and distant metastasis (Fig. 5). HMGB1 was positively expressed in 90 % of cells in all tumor stages and across our selected CRC samples. HMGB1 has been previously implicated in CRC with controversial roles in cancer immunity and metastasis [44–48]. Although, it was not significantly associated with clinic-pathological features in our CRC samples (Fig. 5b), we still observed a consistent mRNA and protein over-expression of HMGB1 (Figs. 1, 5), as well as significant correlation between mRNA and CNV gains (Fig. 3b). Our data suggest that these gene markers can be identified in CRC samples, as early as stage II, but are not solely stage II specific. The consistent overexpression of these gene markers further supports their functional importance as oncogenes in tumor progression.

Our bioinformatics analysis suggests that *HMGB1* and *RPN2* are both targeted by an oncogenic TF, namely NFkB1 (Fig. 2). In fact, aberrant expression of NFkB1 expression was also observed in about 29.7 % of stage III/IV CRC cases (Fig. 5), which is consistent with previously published findings [43]. The *RPN2* gene, located on chromosome 20q13, encodes a proteasome scaffolding protein that inhibits *Bcl*-mediated apoptosis and stabilizes mutated *p53* protein expression through inactivation of *GSK3β* in breast cancer [49, 50]. Over-expression of *RPN2* in stage II CRC was also reported by another microarray that focused on CRC tumor metastasis (Table 1), supporting its implication beyond early staged tumors. In TCGA CRC dataset, *RPN2* up-regulation was observed in 65 out of 195 CRC cases (37 %) and was significantly associated with copy number gain (Fig. 3). This is consistent with previous findings, where >65 % of CRC cases have shown gains on chromosome and a strong association with liver metastasis and poor outcome [51–53]. In addition, we examined RPN2- and HMGB1 alteration profiles in 195 CRC staged patients, available in the TCGA database, by ranking them according to their alteration rates (Additional file 2: Figure S2). Further evaluation revealed a slightly longer survival of patients without RPN2 alteration, but that did not reach statistical significance (p = 0.38, data not shown). This survival data may be valid, because the marker was not originally identified as a predictor for CRC survival in all stage cancers. It will be interesting to complement the genetic alteration and differential expression profiles of these molecules as biomarkers in future clinical trials for stage II CRC patients.

Furthermore, the importance of *RPN2* in tumor prognosis and therapeutic implications has been documented in other solid cancers, including esophageal squamous cell carcinoma [54, 55], osteosarcoma [56, 57] and breast cancer [50]. We observed that *RPN2* is also highly altered in head and neck squamous cell carcinoma (HNSCC), as well as lung squamous cell carcinoma (Fig. 4b), consistent with previously published studies [55, 58]. In various human malignancies, silencing of *RPN2* was associated with increased apoptosis, reduced tumor growth and increased sensitivity of tumor cells to docetaxel response [49, 54]. The value of *RPN2*, both as a prognostic marker and as a therapeutic target is suggested for future validation. In this manuscript, we have shown that CRC cases with RPN2 staining were significantly higher in stage III/IV, in distant metastatic, and poorly differentiated tumors, indicating that its expression is associated with worse prognosis. In addition, NFkB1 protein expression was also associated with distant metastasis. Our data presents experimental evidence that RPN2 protein expression could serve as a potential biomarker to predict metastasis and worse prognosis. However, due to the lack of patient survival and outcome data in the current study, these molecules need to be further validated for their value as biomarkers in larger clinical trials.

## Conclusions

In this manuscript, by utilizing mRNA profiling, we have identified a panel of differentially expressed gene signatures in stage II colorectal adenocarcinoma tissues. Through integrated analyses of the transcriptional regulation, The Cancer Genome Atlas database, and 50 published microarray studies of colorectal cancer specimens, we have identified eight candidate genes that are significantly associated with the aggressive phenotype, including *RPN2*, *HMGB1*, *AARS*, *IGFBP3*, *STAT1*, *HYOU1*, *NQO1* and *PEA15*. Among those genes, *RPN2* and *HMGB1* displayed higher frequencies of genomic alterations in colorectal cancer, compared to other solid tumors. Furthermore, RPN2 protein expression evaluated by immunohistochemistry in 78 independent (stage I–IV) colorectal cancer tissues, exhibited a significant association with stage III/IV tumors, distant metastasis, and poor differentiation. Our study identified important molecular signatures underlying malignant progression and phenotype of colorectal cancer, which warrants future clinical investigations.

## Methods

### Patients

This study was approved by the ethical committee at Inner Mongolia Medical University and a written informed patient consent was obtained. A total of 82 patients were diagnosed with pathological stages (Additional file 2: Figure S3) and underwent surgical resection for CRC at Inner Mongolia Medical University Hospital from 2002 to 2006 and were diagnosed with pathological stages. Patients with hereditary syndromes, e.g. familial adenomatous polyposis (FAP), Lynch syndrome or hereditary nonpolyposis colorectal cancer (HNPCC), or inflammatory syndromes were pre-screened and excluded from this study. The preoperational chemo-radiotherapy or chemotherapy could significantly influence the expression of biomarkers. Hence none of the patients used in this study received treatment prior to surgery. Tumor staging was performed according to TNM classification criteria and guidelines of the International Union Against Cancer (UICC) guidelines [23]. Histological differentiation was evaluated, as poorly differentiated carcinomas are known to have a high-risk of recurrence or metastasis. Accordingly, we randomly selected 4 poorly differentiated adenocarcinoma (stage II) samples with matched adjacent normal mucosa tissues and performed microarray analysis. Histological evaluation confirmed the content of tumor- or normal colon epithelium cells to be more than 50 % (Additional file 2: Figure S1b).

### RNA extraction and microarray profiling analysis

Total RNA was isolated from frozen tissue samples and extracted using the RNeasy Mini Kit (QIAGEN, Maryland, USA) according to manufacturer’s instructions. Gene expression was analyzed using Oligo GEArray Human Cancer Microarray^®^ (Cat# OHS-802SA, Biosciences, CA, USA), which contains a total of 480 probes for 440 genes, encoding for tumor suppressors, oncogenes, signal transduction molecules, growth factors and their corresponding receptors, as well as others associated with angiogenesis. Gene expression levels were normalized to the *beta*-*actin* housekeeping gene. The selection criterion of differentially expressed genes was based on at least 1.5-fold threshold between the CRC tumors and matched normal tissues.

### Computational inference of transcription factor target genes

We have developed a mathematical model, capable to identify target genes of a particular transcription factor (TF) and thus to construct a gene network, regulated by that TF. By recursively applying this model to the identified network, multiple networks can be interconnected. Moreover, the model is able to infer how likely a gene is regulated by a particular TF, which has been successfully applied in other cancer datasets [24–28]. In this study, this model was applied to the above-obtained CRC differential gene expression data, in order to investigate target genes regulated by seven common cancer-related TFs, including NFKB1, RELA, AP1, TP53, TP63, STAT3 and MYC.

### Genomic alterations from TCGA database

The cancer genome Atlas (TCGA) project has published the first “Marker” paper of colon cancer in 2012, which included genomic sequencing, epigenetic and mRNA expression profiling across 195 human colorectal cancer specimens [20]. The data is accessible through the cBio Cancer Genomics Portal (http://cbioportal.org) [29], a web resource designed for the visualization of oncogenomic datasets. Using the differentially expressed gene list in our current study, we extracted genomic alteration profiles from 195 CRC specimens, which have been published and deposited in the TCGA database. Since the first “Marker” paper of colon cancer was published [20], there is a constant submission of more colon cancer samples to the TCGA project, for continued generation of high throughput experimental data. However, not all of these data are complete or have gone through the confirmation and validation process, and thus are not included in this study.

### Immunohistochemical analysis

Thin sections of 10 % formalin-fixed, paraffin-embedded tissue specimens were treated with goat anti-human RPN2 antibody (sc-12165, Santa Cruz), rabbit anti-human HMGB1 antibody (#6893S, Cell Signaling), or rabbit anti-human NFKB1 antibody (sc-1190, Santa Cruz), followed by a peroxidase-conjugated goat anti-rabbit (sc-2018, Santa Cruz) or rabbit anti-goat (sc-2023, Santa Cruz) secondary antibody. Color was developed using Avidin and Biotin-conjugated horseradish peroxidase (ABC reagents) and according to standard protocols. The percentage of positively stained cancer cells was determined under the microscope from more than four visual fields (at 400× magnification). Specimens were evaluated by two independent pathologists and classified into 2 groups: negative staining (no cells were intensely stained), and positive staining (at least 10 % cells were intensely stained) [30].

### Statistical analysis

Correlation between gene expression and distinct clinicopathologic characteristic was analyzed by the Fisher’s exact test. For all statistical analysis, a *P* value of <0.05 was considered significant.

## Additional files

Additional file 1:
**Table S1**. List of differentially expressed genes with transcriptional regulation. **Table S2**. Gene Ontology (GO) and Ingenuity Pathway Analysis (IPA) of differentially expressed genes based on biological processes, canonical pathways and upstream regulators. **a** GO biological processes enriched (P<0.01, FDR<5%). **b** Upstream regulator analysis using Ingenuity Pathway Analysis 2013 (P<0.00001) **Table S3**. Comparison of differentially expressed genes with published CRC microarray studies Additional file 2:
**Figure S1**. Ingenuity Pathway Analysis (IPA) identifies enriched top pathway networks in CRC.**Figure S2**. Oncoprint summary of genomic alterations in CRC from the TCGA database. **Figure S3**. Clininopathological characteristics of CRC samples

## Abbreviations

CRC: colorectal cancer
GO: gene ontology
IPA: ingenuity pathway analysis
TCGA: The Cancer Genome Atlas
CNVs: copy number variations
TF: transcription factor
RPN2: ribophorin II
HMGB1: high-mobility group protein B1
