# Supplementary figures and images for: Integrated transcriptional profiling and genomic analyses reveal RPN2 and HMGB1 as promising biomarkers in colorectal cancer
Source: Cell Biosci. 2015 Sep 17;5:53. doi: 10.1186/s13578-015-0043-9 (PMC4574027; doi:10.1186/s13578-015-0043-9)

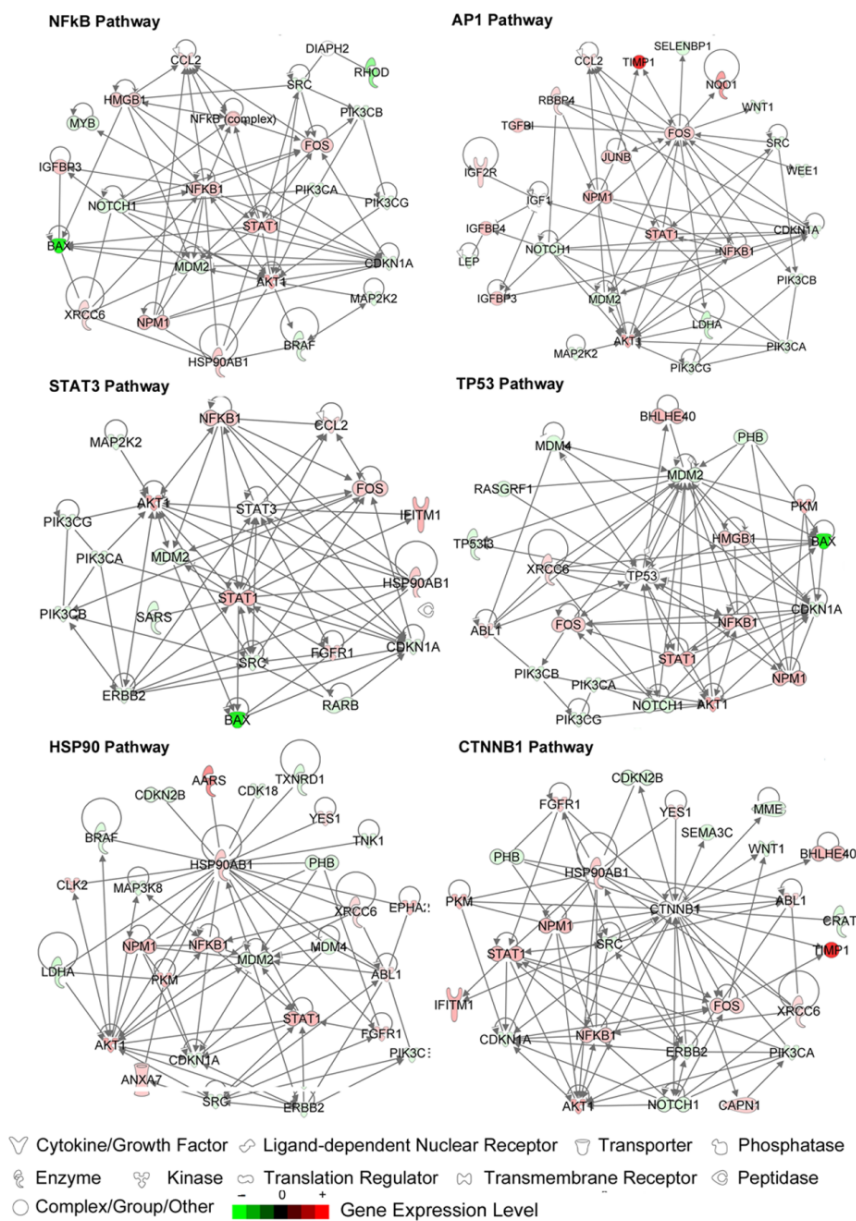

**Supplementary Figure S1**

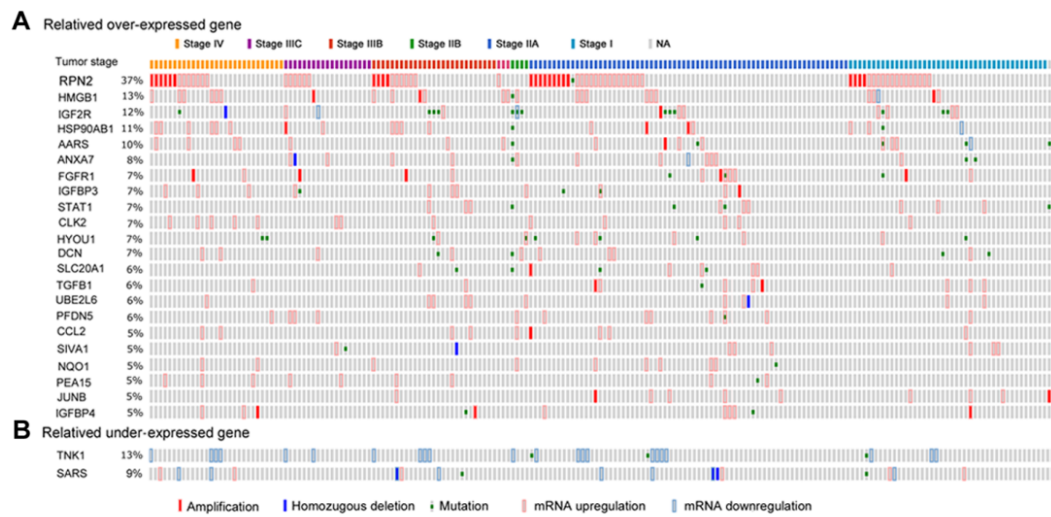

Supplementary Figure S2

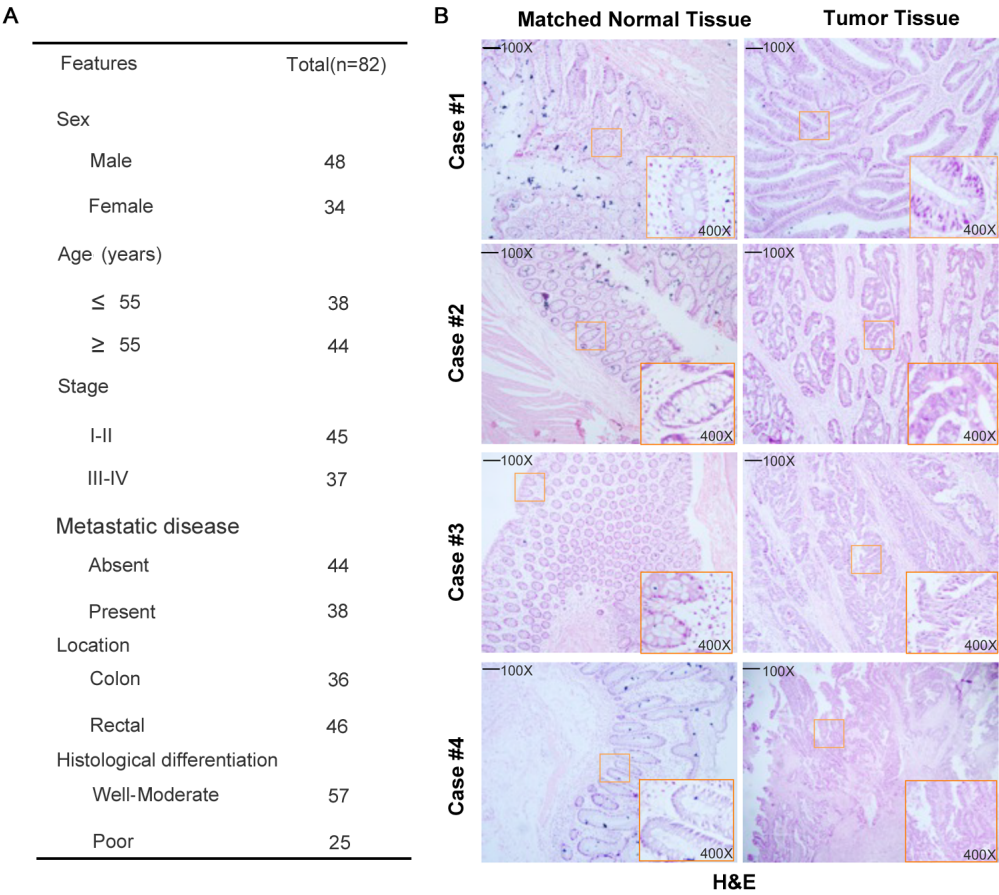

**Supplementary Figure S3**

Supplement: Additional file 2: — Figure S1. Ingenuity Pathway Analysis (IPA) identifies enriched top pathway networks in CRC.Figure S2. Oncoprint summary of genomic alterations in CRC from the TCGA database. Figure S3. Clininopathological characteristics of CRC samples [file 13578_2015_43_MOESM2_ESM.pdf]
